# Pharmacokinetics and Exploratory Exposure–Response Analysis of Chikusetsusaponin IVa in Myocardial Ischemia/Reperfusion-Injured Rats

**DOI:** 10.3390/ph19050749

**Published:** 2026-05-11

**Authors:** Xiaomin Shuai, Hui Wang, Jianmin Luo, Yangqiao Zeng, Ying Wang, Lijun Zhu, Zhongqiu Liu, Yuanyuan Cheng

**Affiliations:** State Key Laboratory of Traditional Chinese Medicine Syndrome, International Institute for Translational Chinese Medicine, School of Pharmaceutical Science, Guangzhou University of Chinese Medicine, Guangzhou 510006, China

**Keywords:** Chikusetsusaponin IVa, myocardial ischemia-reperfusion injury, pharmacokinetics, exploratory exposure–response analysis, concentration–effect relationship

## Abstract

**Background:** Myocardial ischemia/reperfusion injury (MIRI) remains a major limitation to effective cardioprotection. Chikusetsusaponin IVa (CS-IVa) has shown promising cardioprotective activity; however, its pharmacokinetic behavior and exposure–response relationship under MIRI pathological conditions remain insufficiently characterized. This study aimed to evaluate the disease-state-related pharmacokinetics of CS-IVa in MIRI rats and to explore its concentration–effect relationship using a revised descriptive PK framework. **Methods:** A rat MIRI model was established by ligation and reperfusion of the left anterior descending coronary artery. The cardioprotective effects of CS-IVa were evaluated using echocardiography, hemodynamic parameters, myocardial infarct size, histopathological examination, and biochemical markers of myocardial injury and oxidative stress. Plasma CS-IVa concentrations were quantified by UHPLC-MS/MS over 0–24 h after administration. Non-compartmental pharmacokinetic parameters were statistically compared between normal and MIRI rats. To address model reliability and parameter identifiability, candidate PK models with different structural assumptions and weighting schemes were systematically re-evaluated. The selected descriptive PK model was further assessed using the leave-one-rat-out robustness analysis. An exploratory exposure–response analysis was performed using CK-MB as the longitudinal PD endpoint, and a Ke0 sensitivity analysis was conducted to evaluate the robustness of the downstream effect-compartment interpretation. Data-driven models were retained only as supplementary exploratory predictive analyses. **Results:** CS-IVa improved cardiac function; reduced myocardial infarct size; attenuated histopathological injury; decreased serum CK-MB, cTnI, LDH and plasma MDA levels; and restored SOD activity in MIRI rats. In normal rats, systemic exposure to CS-IVa increased with dose escalation. Compared with normal rats at 15 mg/kg, MIRI rats showed markedly altered pharmacokinetic behavior, including reduced C_max_ and AUC, delayed T_max_, shortened apparent half-life, and increased apparent volume of distribution. After systematic model re-evaluation, a one-compartment model with first-order absorption, no lag time, and unweighted fitting was selected as the revised working descriptive PK model, providing a better balance between model fit, parameter stability, and parsimony. The leave-one-rat-out analysis supported the robustness of this revised model. The exploratory concentration–effect analysis revealed a temporal dissociation between plasma CS-IVa exposure and CK-MB response, suggesting a delayed pharmacodynamic response. Ke0 sensitivity analysis indicated that effect-compartment-based PD fitting was sensitive to Ke0 selection; accordingly, the exposure–response analysis is interpreted as exploratory rather than as a definitive mechanistic PK/PD model. **Conclusions:** CS-IVa exerted cardioprotective effects in MIRI rats, while MIRI markedly altered its overall pharmacokinetic behavior. The revised analysis supports disease-state-related PK changes and an exploratory exposure–response delay between plasma CS-IVa exposure and CK-MB response. These findings provide a pharmacokinetic basis for understanding CS-IVa under MIRI pathological conditions; however, further studies incorporating individual-level PD endpoints, tissue distribution data, and clinically relevant formulations are needed before translational dosing recommendations can be made.

## 1. Introduction

Acute myocardial infarction (AMI) remains one of the leading causes of cardiovascular mortality and morbidity worldwide [1]. Timely restoration of coronary perfusion to the ischemic myocardium represents the cornerstone therapeutic strategy for improving clinical outcomes [2]. However, while reperfusion is essential for salvaging jeopardized cardiomyocytes, it paradoxically induces myocardial ischemia/reperfusion injury, further exacerbating myocardial damage through oxidative stress, inflammatory activation, mitochondrial dysfunction, calcium overload, endothelial injury, and apoptosis [3,4]. Consequently, attenuation of MIRI has emerged as a critical scientific challenge for maximizing the clinical benefit of reperfusion therapy and advancing the translational application of cardioprotective strategies.

Although numerous candidate agents have demonstrated cardioprotective effects in experimental MIRI models, their clinical translation has remained largely unsatisfactory [5]. One important contributing factor is that existing studies have predominantly focused on endpoint efficacy measures-such as infarct size, myocardial enzyme profiles, and cardiac function-while affording insufficient attention to the in vivo exposure characteristics of drugs under pathological conditions and the dynamic quantitative relationship between drug exposure and pharmacodynamic response [6]. For agents intended for acute ischemia/reperfusion intervention, therapeutic outcomes depend not only on intrinsic pharmacological activity but also on whether adequate drug exposure can be achieved and sustained within the critical therapeutic time window; moreover, the pathological state itself may substantially alter drug absorption, distribution, metabolism, and excretion, thereby influencing true therapeutic efficacy [7]. Therefore, evaluating disease-state-related pharmacokinetic changes and exploratory exposure–response relationships is important for understanding the in vivo behavior of cardioprotective candidates under MIRI conditions.

**Figure 1:**
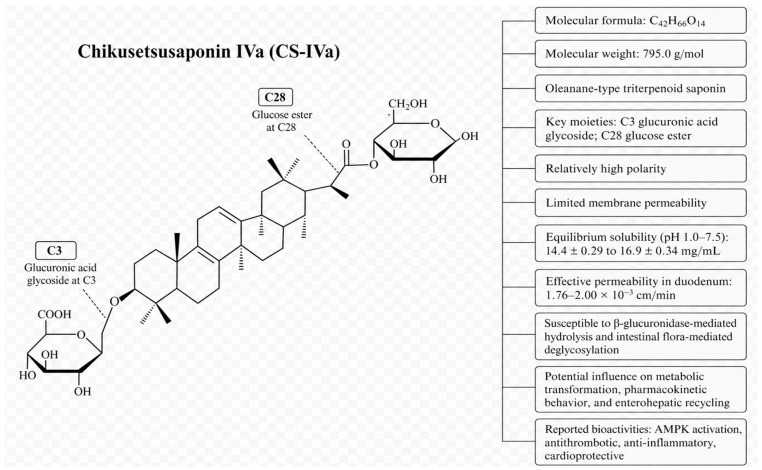
Chemical structure and key physicochemical features of Chikusetsusaponin IVa (CS-IVa; C_42_H_66_O_14_; MW 795.0 g/mol). The glucuronic acid glycoside moiety at C3 and glucose ester moiety at C28 confer high polarity and limited membrane permeability, and may influence its metabolic transformation, pharmacokinetic behavior, and potential enterohepatic recycling.

Chikusetsusaponin IVa is an oleanane-type pentacyclic triterpenoid saponin derived primarily from *Panax japonicus*, *Panax japonicus var. major*, and related Araliaceae species, representing one of their characteristic bioactive constituents [8]. Structurally, CS-IVa has the molecular formula C_42_H_66_O_14_ and a molecular weight of approximately 795.0 g/mol. It contains a glucuronic acid glycoside moiety at C3 and a glucose ester moiety at C28, conferring relatively high polarity and amphiphilic properties. These physicochemical features may influence membrane permeability, tissue distribution, metabolic transformation, and systemic exposure, and may also render CS-IVa susceptible to *β*-glucuronidase-mediated hydrolysis and intestinal flora-mediated deglycosylation. The chemical structure of CS-IVa is shown in Figure 1.

Pharmacologically, CS-IVa has been reported to possess anti-inflammatory, antioxidant, antithrombotic, and cytoprotective properties, and to attenuate myocardial injury through pathways related to AMPK activation, NF-*κ*B inhibition, and Nrf2/HO-1 signaling regulation [9,10]. In ischemic and ischemia/reperfusion injury models, CS-IVa has also shown the ability to reduce tissue injury and improve functional outcomes [11]. Nevertheless, existing research on CS-IVa has largely remained at the level of pharmacodynamic endpoint assessment. Whether its pharmacokinetic behavior is altered under the pathological conditions of MIRI, whether systemic exposure differs between normal and pathological states, and how plasma exposure relates temporally to myocardial injury biomarkers remain insufficiently characterized.

MIRI is accompanied by a series of pathophysiological alterations, including abnormal tissue perfusion, endothelial dysfunction, inflammatory activation, oxidative stress, tissue acidosis, and potential changes in metabolic enzyme and transporter activity [12,13]. These changes may interact with the physicochemical properties of CS-IVa and further affect its in vivo disposition. Therefore, when evaluating the cardioprotective effects of CS-IVa, comparative studies under both normal and MIRI conditions are warranted to delineate the impact of the pathological state on its pharmacokinetic behavior. On the other hand, drug concentration and pharmacodynamic response are frequently asynchronous, particularly when drug distribution to the effect site is slow or when the pharmacodynamic effect is dependent on downstream signal transduction and biological responses; under such circumstances, the pharmacodynamic response may lag behind changes in plasma concentration, manifesting as a hysteresis loop in the concentration-effect relationship [14,15]. Such temporal dissociation is particularly relevant when injury-release biomarkers such as CK-MB are used as pharmacodynamic endpoints.

Quantitative pharmacokinetic analysis combined with exploratory exposure–response assessment provides a useful framework for investigating drug behavior under pathological conditions [16]. Non-compartmental analysis can directly compare systemic exposure between normal and disease states, whereas descriptive compartmental models can provide smoothed concentration–time profiles for subsequent exploratory concentration–effect assessment. Effect-compartment approaches have been widely employed to empirically describe temporal dissociation between plasma concentration and pharmacodynamic response and represent one of the classical approaches for characterizing concentration–effect asynchrony [17,18]. However, because biomarker-based exposure–response analysis may be sensitive to model structure, parameter identifiability, and empirical delay assumptions, model selection should consider not only goodness-of-fit but also numerical stability, parameter precision, condition number, residual behavior, and parsimony. Furthermore, given that pharmacodynamic changes under pathological conditions may exhibit pronounced nonlinearity and time-dependence, data-driven approaches such as deep learning may provide supplementary predictive insights for complex exposure–response data, but their interpretation should remain exploratory, particularly when sample size is limited, and graph structures are computational rather than biological [19,20].

On this basis, the present study evaluated the cardioprotective effects of CS-IVa in a rat model of MIRI and investigated its pharmacokinetic behavior under normal and pathological conditions. Specifically, we aimed to: (i) assess the cardioprotective effects of CS-IVa using cardiac functional, histopathological, biochemical, and oxidative stress-related indicators; (ii) compare non-compartmental pharmacokinetic parameters between normal and MIRI rats; (iii) re-evaluate candidate PK models and select a numerically stable descriptive PK model for CS-IVa exposure characterization; and (iv) perform exploratory exposure–response analysis using CK-MB as the longitudinal pharmacodynamic endpoint. Deep learning models were retained only as supplementary exploratory predictive analyses and were not used to support mechanistic PK/PD conclusions. This revised framework was intended to provide a pharmacokinetic basis for understanding CS-IVa behavior under MIRI pathological conditions while avoiding overinterpretation of exploratory modeling results.

## 2. Results

### 2.1. CS-IVa Protects Against MIRI

#### 2.1.1. CS-IVa Improves Cardiac Function

**Figure 2:**
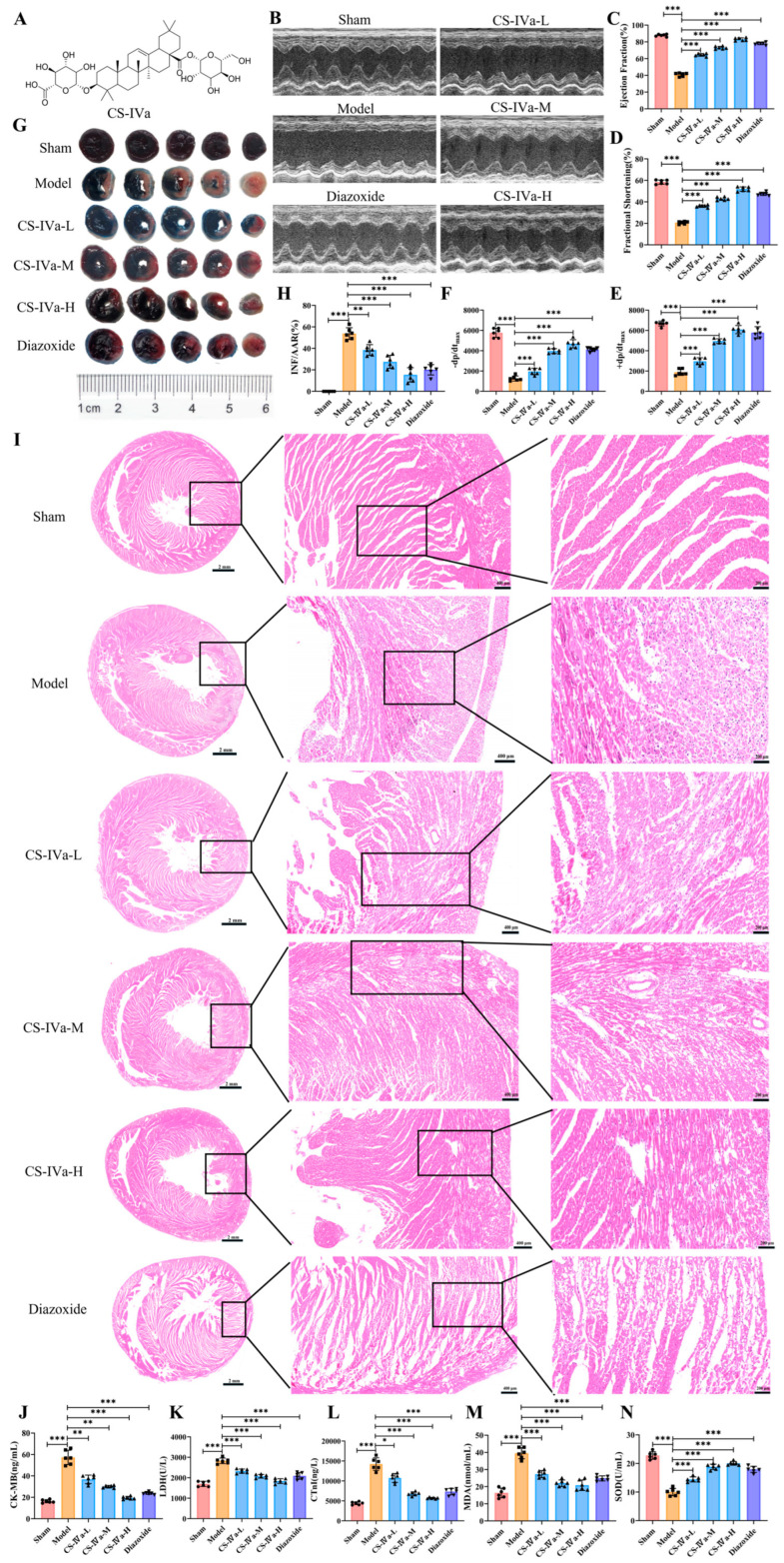
CS-IVa attenuates myocardial ischemia/reperfusion injury in rats: (**A**) Chemical structure of CS-IVa. (**B**) Representative M-mode echocardiographic images. (**C**–**F**) Quantitative analysis of left ventricular ejection fraction (LVEF), left ventricular fractional shortening (LVFS), maximum rate of left ventricular pressure rise (+dp/dt_max_), and maximum rate of left ventricular pressure decline (-dp/dt_max_) at 24 h after MIRI. (**G**,**H**) Representative Evans Blue/TTC staining images and quantitative analysis of myocardial infarct size. (**I**) Representative H&E-stained myocardial sections. Scale bars: 2 mm, 400 μm, and 200 μm. (**J**–**N**) Serum CK-MB, LDH, cTnI, and MDA levels and serum SOD activity in each group. Data are presented as mean ± SD (*n* = 6). Statistical significance was determined by one-way ANOVA followed by Bonferroni’s post hoc test. * *p* < 0.05, ** *p* < 0.01, *** *p* < 0.001.

Cardiac function was evaluated by echocardiography and hemodynamic assessment at 24 h after ischemia/reperfusion (Figure 2B–F). Compared with the sham group, the MIRI group exhibited marked left ventricular dysfunction, manifested as significant reductions in LVEF, LVFS, +dp/dt_max_, and −dp/dt_max_. Compared with the MIRI group, CS-IVa treatment significantly improved all these functional parameters, indicating that it restored impaired left ventricular systolic and diastolic function following ischemia/reperfusion. The most pronounced improvements were observed in the medium- and high-dose groups, consistent with the trends shown in the representative M-mode echocardiographic images.

#### 2.1.2. CS-IVa Reduces Myocardial Injury

Evans Blue/TTC staining and histopathological examination further confirmed the protective effects of CS-IVa. Evans Blue/TTC double staining revealed that, compared with the sham group, the MIRI group displayed a significantly larger myocardial infarct size (Figure 2G,H). Treatment with CS-IVa markedly reduced infarct size in a dose-related trend, with the protection afforded by the high-dose group approaching that of the diazoxide group. Histopathological findings were consistent with these results (Figure 2I). Myocardial tissue in the sham group showed intact architecture with tightly and regularly arranged myofibers and no evident inflammatory cell infiltration. In contrast, the MIRI group exhibited pronounced structural disruption, including loosely arranged myofibers, widened intercellular spaces, vacuolar degeneration, and inflammatory cell infiltration. These pathological changes were markedly attenuated by CS-IVa treatment, particularly in the medium- and high-dose groups, which showed more regular myocardial arrangement and reduced degrees of tissue injury.

#### 2.1.3. CS-IVa Alleviates Biochemical Damage

Serum biochemical parameters further supported the cardioprotective effects of CS-IVa (Figure 2J–N). Compared with the sham group, MIRI significantly elevated Serum CK-MB, LDH, cTnI, and MDA levels and serum SOD activity. CS-IVa treatment markedly reversed these changes, as evidenced by decreased levels of CK-MB, LDH, cTnI, and MDA alongside restoration of SOD activity. These results indicate that CS-IVa is capable of alleviating myocardial injury and oxidative stress following ischemia/reperfusion.

### 2.2. UHPLC-MS/MS Method Validation

**Figure 3:**
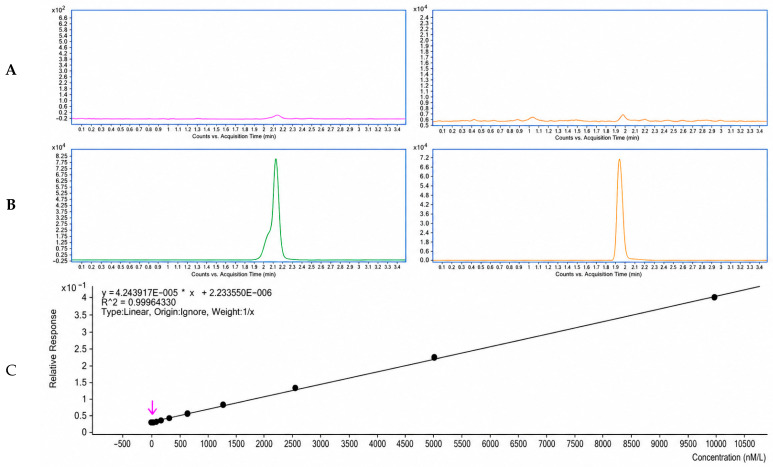
MRM chromatograms and calibration curve for CS-IVa quantification in rat plasma: (**A**) Blank plasma. (**B**) Reference solution containing CS-IVa and the internal standard. (**C**) Blank plasma spiked with CS-IVa and the internal standard.

The developed UHPLC-MS/MS method enabled reliable and selective quantification of CS-IVa in rat plasma. Representative MRM chromatograms showed no significant endogenous interference at the retention times of CS-IVa or the internal standard (Figure 3A–C). The calibration curve exhibited good linearity over the concentration range of 1.22–10,000 nM, with the regression equation Y = 0.000042X + 0.000002 and R^2^ = 0.9996. The lower limit of quantification was 1.22 nM (Table 1). Precision, accuracy, matrix effect, extraction recovery, stability, and dilution integrity all met acceptable analytical criteria, confirming that the method was suitable for pharmacokinetic analysis of CS-IVa in rat plasma. Detailed validation results are summarized in Tables S2–S5.

### 2.3. Pharmacokinetics and Exploratory Exposure–Response of CS-IVa

#### 2.3.1. Pharmacokinetic Profile of CS-IVa in Normal Rats

**Figure 4:**
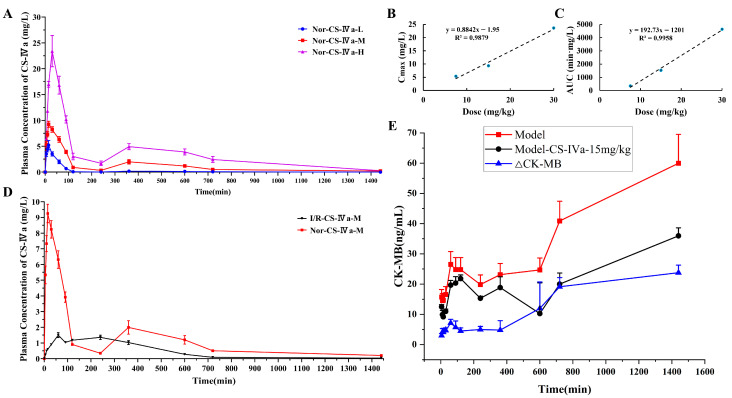
Pharmacokinetic characteristics and exploratory CK-MB response of CS-IVa in rats: (**A**) Plasma concentration–time curves of CS-IVa in normal rats (Nor-CS-IVa-L, Nor-CS-IVa-M, and Nor-CS-IVa-H represent 7.5, 15, and 30 mg/kg, respectively) after intraperitoneal administration. (**B**,**C**) Exploratory regression analyses of dose versus C_max_ (R^2^ = 0.9879) and AUC_0–t_ (R^2^ = 0.9958) in normal rats. (**D**) Plasma concentration–time curves of CS-IVa at 15 mg/kg in normal (Nor-CS-IVa-M) and MI/RI rats (I/R-CS-IVa-M). (**E**) Time-course profiles of CK-MB in the untreated MI/RI group (Model) and CS-IVa-treated MI/RI group (Model-CS-IVa-15 mg/kg), together with the exploratory ΔCK-MB response curve. ΔCK-MB was calculated as the CK-MB level in the untreated MI/RI group minus the CK-MB level in the CS-IVa-treated MI/RI group at each matched time point.

Following intraperitoneal administration in normal rats, CS-IVa was rapidly absorbed and systemic exposure increased with dose escalation. As shown in Table 2, C_max_ and AUC_0–t_ increased markedly from 7.5 to 30 mg/kg. Exploratory regression analysis confirmed positive dose–exposure associations, with R^2^ = 0.9879 for dose versus C_max_ and R^2^ = 0.9958 for dose versus AUC_0–t_ (Figure 4A–C). A modest secondary rise in plasma concentration was observed at approximately 6 h in the medium- and high-dose groups, suggesting potential enterohepatic recycling or multiphasic absorption. Overall, CS-IVa exhibited a dose-related increase in systemic exposure in normal rats.

#### 2.3.2. MIRI Alters the Pharmacokinetic Behavior of CS-IVa

At 15 mg/kg, marked pharmacokinetic differences were observed between normal and MI/RI rats (Figure 4D, Table 2). Compared with the Nor-15 mg/kg group, the MI/RI-15 mg/kg group showed significantly reduced C_max_ and AUC, delayed T_max_, shortened apparent terminal half-life, and increased apparent volume of distribution (all *p* < 0.001). MRT_0−∞_ was also reduced (*p* = 0.004), while MRT_0−t_ did not differ significantly. Apparent clearance was descriptively higher in the MI/RI group, but is reported without statistical comparison due to rounding of summarized values. These findings indicate that MIRI markedly altered the apparent pharmacokinetic behavior of CS-IVa, particularly systemic exposure, absorption, and apparent distribution.

#### 2.3.3. CK-MB Time Course and Exploratory ΔCK-MB Response

The CK-MB time course was used to characterize the exploratory pharmacodynamic response to CS-IVa. As shown in Figure 4E, CK-MB levels in the untreated MI/RI group (Model) remained consistently higher than those in the CS-IVa-treated MI/RI group (Model-CS-IVa-15 mg/kg) across all measured time points. The ΔCK-MB response showed limited change at early time points but progressively increased during the later post-dose period, suggesting that the CK-MB response lagged behind plasma CS-IVa exposure and providing the basis for subsequent exploratory hysteresis analysis.

### 2.4. Revised PK Model Selection and Robustness Analysis

To address model reliability and parameter identifiability, candidate PK models for CS-IVa in MI/RI rats were systematically re-evaluated. One-compartment and two-compartment models with first-order absorption were compared, with or without lag time, under three weighting schemes: unweighted, 1/Ŷ, and 1/Ŷ^2^, where Ŷ represents the model-predicted concentration. Model selection was based not only on goodness-of-fit diagnostics and information criteria, but also on parameter precision, condition number, convergence behavior, residual distribution, and model parsimony. The complete candidate model comparison is provided in Supplementary Table S6.

**Figure 5:**
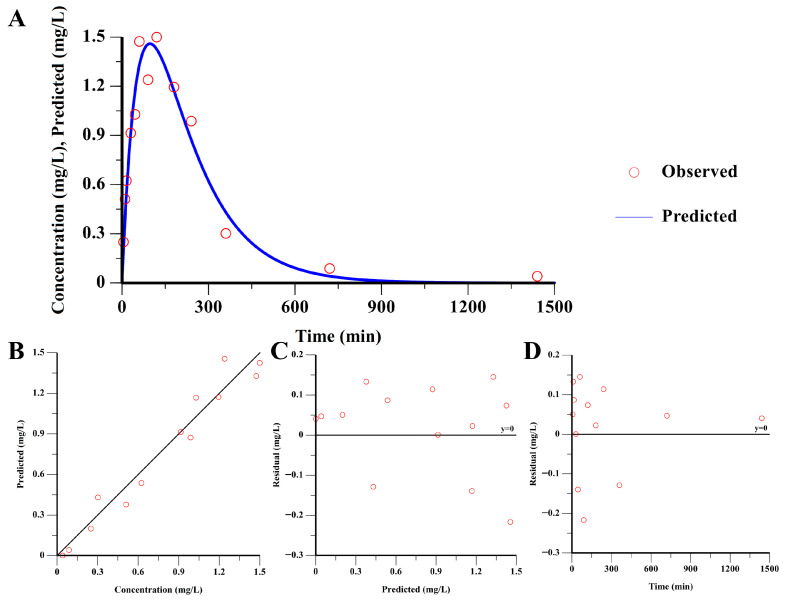
Goodness-of-fit diagnostics for the revised descriptive PK model of CS-IVa in MI/RI rats: (**A**) Observed and model-predicted plasma concentration–time profiles. (**B**) Observed versus predicted concentrations. (**C**) Residuals versus predicted concentrations. (**D**) Residuals versus time.

The one-compartment model with first-order absorption, no lag time, and unweighted fitting was selected as the revised working descriptive PK model. This model converged successfully and yielded AIC = −18.274, SBC = −16.579, and a condition number of 1518, demonstrating improved numerical stability and more acceptable parameter identifiability for descriptive purposes compared with more complex candidate models. Goodness-of-fit diagnostics confirmed acceptable descriptive performance and residual behavior (Figure 5).

To further assess model robustness, leave-one-rat-out refitting was performed in the MI/RI-15 mg/kg group. All refits converged successfully. The condition number remained within 1067–2455, predicted C_max_ ranged from 1.3843 to 1.5364 mg/L, predicted T_max_ ranged from 93.78 to 102.23 min, and predicted AUC ranged from 410.64 to 431.11 min·mg/L. These results indicate that the revised descriptive PK model was not disproportionately driven by any single animal. Detailed robustness results are provided in Supplementary Table S7.

### 2.5. Exploratory Exposure–Response Analysis

**Figure 6:**
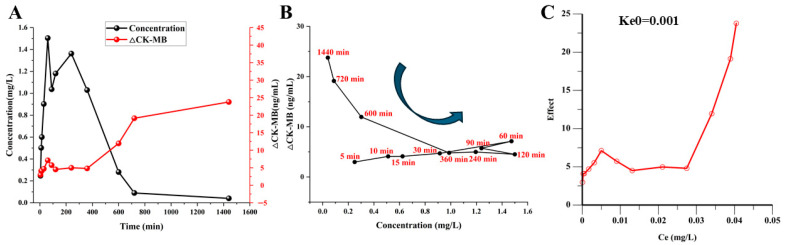
Exploratory exposure–response analysis of CS-IVa in MI/RI rats: (**A**) Dual-axis time-course profiles of plasma CS-IVa concentration (left axis, mg/L) and ΔCK-MB response (right axis, ng/mL). (**B**) Plasma concentration–ΔCK-MB hysteresis loop showing counterclockwise temporal dissociation, with time points labeled at key intervals. (**C**) Representative exploratory effect-compartment concentration–ΔCK-MB plot at Ke0 = 0.001 min^−1^. Ke0 = 0.001 min^−1^ was selected as the representative empirical value among successfully converged candidate values, while the overall effect-compartment analysis was interpreted as exploratory rather than as a definitive mechanistic PK/PD model.

To explore the temporal relationship between plasma CS-IVa exposure and ΔCK-MB response, concentration–effect plots were constructed using plasma concentration and ΔCK-MB values at matched time points. Plasma CS-IVa concentration increased rapidly after intraperitoneal administration and then declined, whereas ΔCK-MB showed a delayed increase during the later post-dose period (Figure 6A). This temporal dissociation was further reflected by a counterclockwise hysteresis pattern in the plasma concentration–ΔCK-MB plot (Figure 6B), indicating that plasma concentration alone did not directly correspond to the observed ΔCK-MB response.

An effect-compartment approach was applied as an empirical exploratory method to describe this temporal delay. Ke0 sensitivity analysis showed that downstream PD fitting was sensitive to Ke0 selection, with fitting failures observed at Ke0 values of 0.0002, 0.0001, and 0.00005 min^−1^. Among the successfully converged candidate Ke0 values, Ke0 = 0.001 min^−1^ showed the best overall fitting performance, with SSR = 342.703, AIC = 79.879, CORR = 0.565, and a normalized hysteresis loop area of 0.193. A representative exploratory effect-compartment concentration–ΔCK-MB plot at Ke0 = 0.001 min^−1^ is shown in Figure 6C. These findings indicate that the effect-compartment analysis should be interpreted only as an exploratory empirical description of the temporal dissociation between plasma CS-IVa exposure and ΔCK-MB response, rather than as a definitive mechanistic PK/PD model. Complete Ke0 sensitivity results are provided in Supplementary Table S8 and Supplementary Figure S2.

As supplementary exploratory predictive analyses, three data-driven models—GAT, LSTM, and MLP—were evaluated for ΔCK-MB response prediction. The input feature was normalized plasma CS-IVa concentration, and the output variable was the ΔCK-MB response. The graph representation in the GAT model was defined as a computational association graph for PK–PD observations rather than a biological molecular interaction network. These models were used only as supplementary predictive tools and were not used for mechanistic inference or to support primary PK/PD conclusions. Detailed model architectures, training parameters, and prediction results are provided in Supplementary Methods S8 and Supplementary Figure S4.

## 3. Discussion

The present study demonstrated that CS-IVa exerted cardioprotective effects in a rat model of myocardial ischemia/reperfusion injury, as reflected by improved cardiac function, reduced infarct size, attenuated histopathological injury, decreased myocardial injury biomarkers, and improved oxidative stress-related indices. In parallel, the pathological state of MI/RI markedly altered the pharmacokinetic behavior of CS-IVa, with reduced systemic exposure and delayed absorption-related parameters compared with normal rats at the same dose. The revised analytical strategy further indicated a temporal dissociation between plasma CS-IVa exposure and CK-MB-derived response. Importantly, this exposure–response analysis was interpreted as exploratory rather than as a definitive mechanistic PK/PD model, given the biomarker-based nature of the PD endpoint and the sensitivity of the effect-compartment analysis to Ke0 selection.

### 3.1. Cardioprotective Effects of CS-IVa Against MIRI

CS-IVa significantly improved left ventricular systolic and diastolic function, reduced myocardial infarct size, alleviated histopathological damage, and attenuated myocardial injury and oxidative stress markers in MI/RI rats. Stronger protective effects were generally observed in the medium- and high-dose groups, and the effect of high-dose CS-IVa approached that of the positive control diazoxide. These findings are consistent with previously reported cardioprotective activity of CS-IVa, including its involvement in SIRT1/ERK1/2 and Homer1a signaling [21], NF-κB-mediated inflammatory inhibition [22], and cell survival signaling pathways related to AMPK activation [9]. The reduction in CK-MB, cTnI, LDH, and MDA levels, together with restoration of SOD activity, suggests that CS-IVa may attenuate myocardial injury partly by reducing reperfusion-associated oxidative stress and secondary inflammatory injury. These pharmacodynamic findings provide the biological basis for further investigating the pharmacokinetic behavior and exploratory exposure–response relationship of CS-IVa under MI/RI pathological conditions.

### 3.2. Disease-State-Related Pharmacokinetic Alteration and Physicochemical Considerations

In normal rats, CS-IVa showed a dose-related increase in systemic exposure, with C_max_ and AUC increasing as the dose increased from 7.5 to 30 mg/kg. However, at the same dose of 15 mg/kg, MI/RI rats showed substantially altered pharmacokinetic behavior compared with normal rats, including lower C_max_ and AUC, delayed T_max_, shortened apparent terminal half-life, and altered apparent distribution. These results indicate that the MI/RI pathological state can markedly influence the in vivo disposition of CS-IVa, consistent with the principle that pathological conditions can alter drug absorption, distribution, metabolism, and elimination [23,24,25]. Pharmacokinetic parameters obtained under normal physiological conditions should therefore not be directly extrapolated to pathological conditions.

The altered PK behavior of CS-IVa under MI/RI conditions may be related to the interaction between disease-induced physiological changes and the physicochemical properties of CS-IVa. CS-IVa is an oleanane-type triterpenoid saponin with a relatively large molecular size, high polarity, and amphiphilic structure. It contains a glucuronic acid glycoside moiety at C3 and a glucose ester moiety at C28, which may influence membrane permeability, tissue distribution, metabolic transformation, and systemic exposure [26]. Under MI/RI conditions, systemic inflammation, oxidative stress, endothelial dysfunction, tissue acidosis, and altered regional perfusion may affect apparent distribution, plasma protein binding, tissue partitioning, and clearance-related processes [7,27]. In particular, MI/RI-associated inflammatory mediators may suppress cytochrome P450 enzyme activity and alter hepatic clearance, as previously demonstrated in ischemia/reperfusion settings [28]. These factors may collectively contribute to the reduced systemic exposure and altered apparent volume of distribution observed in MI/RI rats.

A modest secondary rise in the concentration–time profile was observed, particularly in the medium- and high-dose groups. The glucuronic acid glycoside moiety at C3 may render CS-IVa susceptible to *β*-glucuronidase-mediated hydrolysis, while intestinal flora-mediated deglycosylation may also contribute to its metabolic transformation [26]. These processes, which are well-characterized mechanisms for enterohepatic recycling of glucuronide-containing compounds [29], could potentially be related to secondary plasma exposure. However, because bile excretion, fecal excretion, tissue distribution, and intestinal metabolism data were not collected in the present study, this explanation should be considered a plausible hypothesis rather than a definitive conclusion.

### 3.3. Exploratory Exposure–Response Interpretation of the CK-MB Delay

A counterclockwise hysteresis pattern was observed between plasma CS-IVa concentration and ΔCK-MB response, suggesting a temporal dissociation between systemic exposure and the CK-MB-derived pharmacodynamic response. Such a pattern may reflect delayed distribution to the effect site, downstream biological processes, or the intrinsic release and clearance kinetics of the biomarker itself [14,30]. In the present study, CK-MB was selected as the longitudinal PD endpoint because serial CK-MB measurements were available at time points corresponding to PK sampling. Nevertheless, CK-MB is an injury-release biomarker rather than a direct pharmacological target of CS-IVa. Its serum concentration is determined by cardiomyocyte injury, enzyme release, and systemic clearance [31,32,33], and therefore may not change synchronously with plasma drug concentration.

For this reason, the effect-compartment analysis was used only as an empirical exploratory method to describe the temporal delay between plasma CS-IVa exposure and ΔCK-MB response. The Ke0 sensitivity analysis showed that downstream PD fitting was sensitive to Ke0 selection. Among the successfully converged candidate Ke0 values, Ke0 = 0.001 min^−1^ showed the best overall fitting performance and was selected as a representative empirical value. However, fitting failed at several lower Ke0 values, and residual hysteresis remained. Therefore, the effect-compartment result should not be interpreted as a definitive mechanistic PK/PD model or as a fully identifiable effect-site equilibration process [30].

These findings highlight an important methodological consideration for PK/PD analysis using myocardial injury biomarkers. When the PD endpoint is governed by biomarker turnover, release kinetics, and systemic elimination, conventional effect-compartment models may provide only a limited empirical description. Future studies should consider indirect response models that explicitly incorporate biomarker production and elimination processes [34,35,36,37], or alternatively employ real-time pharmacodynamic endpoints more directly linked to CS-IVa action.

### 3.4. Supplementary Predictive Value of Data-Driven Models

In addition to the revised descriptive PK and exploratory exposure–response analyses, GAT, LSTM, and MLP models were evaluated as supplementary data-driven predictive approaches. The graph attention mechanism used in the GAT model [38] and the recurrent architecture of LSTM [39] have shown utility in capturing complex nonlinear patterns in biomedical data [40,41]. The graph representation in the GAT model was defined as a computational association graph for PK–PD observations rather than a biological molecular interaction network. Therefore, these models were used only for supplementary prediction and were not used for mechanistic inference or to support primary PK/PD conclusions. Given the limited sample size, group-level PD response, and simplified input structure, the data-driven results should be interpreted cautiously and require validation in larger individual-level PK/PD datasets.

### 3.5. Limitations and Translational Considerations

This study has several limitations. First, the CK-MB-derived PD response was based on group-level time courses rather than continuous individual-level PK/PD observations, which limited the precision of exposure–response modeling. Future studies should collect paired individual-level PK and PD data and include additional longitudinal cardiac biomarkers, such as cTnI and LDH, to strengthen exposure–response characterization. Second, although the present study showed that MI/RI markedly altered the pharmacokinetic behavior of CS-IVa, the mechanisms underlying these changes were not directly verified. Potential contributors, including altered tissue perfusion, plasma protein binding, endothelial permeability, transporter function, metabolic enzyme activity, biliary excretion, and fecal excretion, require further experimental investigation.

The translational relevance of the present findings also requires cautious interpretation. Rats and humans differ substantially in cardiovascular physiology, drug metabolism, plasma protein binding, and elimination pathways [42,43]. The surgically induced rat MI/RI model produces a relatively controlled acute injury, whereas human myocardial infarction is highly heterogeneous in infarct location, reperfusion timing, comorbidities, concomitant medications, and baseline organ function [44,45]. The poor translation of preclinical cardioprotective findings to clinical benefit is a widely recognized challenge [46,47], partly because young, drug-naive animal models do not fully reflect the complexity of human patients with comorbidities and polypharmacy [44]. In addition, CS-IVa was administered intraperitoneally in the present study, whereas clinically relevant formulations and administration routes remain to be established. Therefore, the present findings provide a pharmacokinetic and exploratory exposure–response basis for understanding CS-IVa under MI/RI conditions. However, further studies incorporating clinically relevant formulations, individual-level PD endpoints, tissue distribution, safety evaluation, and translational PK/PD bridging are required before dosing recommendations can be made.

## 4. Materials and Methods

### 4.1. Instruments and Reagents

Chikusetsusaponin IVa (CS-IVa, purity > 98%; Chengdu Push Biotechnology Co., Ltd., Chengdu, China) was used as the analyte, and digoxin (purity > 99.7%; MedChemExpress, Monmouth Junction, NJ, USA) was used as the internal standard for UHPLC-MS/MS analysis. Diazoxide (Aladdin Biochemical Technology Co., Ltd., Shanghai, China) was used as the positive control drug. Methanol and acetonitrile of chromatographic grade were used for chromatographic analysis and sample preparation. Dimethyl sulfoxide (DMSO) and sterile normal saline were used for drug dissolution and dilution. Serum CK-MB, cTnI, LDH, MDA, and SOD levels were determined using commercial assay kits according to the manufacturers’ instructions.

Plasma CS-IVa concentrations were quantified using an Agilent 1290 ultra-high-performance liquid chromatography system coupled with an Agilent 6490 triple quadrupole tandem mass spectrometer equipped with an electrospray ionization source (Agilent Technologies, Santa Clara, CA, USA). Chromatographic separation was performed on a Waters ACQUITY UPLC BEH C18 column (2.1 × 100 mm, 1.7 μm; Waters, Milford, MA, USA). Detailed reagent information and analytical conditions are provided in Supplementary Methods S1 and S2.

### 4.2. Animals and Establishment of the MIRI Model

Specific pathogen-free male Sprague-Dawley rats weighing 200 ± 20 g were housed under standard laboratory conditions with free access to food and water. All animal experimental procedures were approved by the Laboratory Animal Ethics Committee of Guangzhou University of Chinese Medicine (approval numbers: 20230104 and 20230313).

The MIRI model was established by transient ligation of the left anterior descending coronary artery for 30 min, followed by reperfusion for 24 h. Rats in the sham-operated group underwent the same surgical procedures without coronary artery ligation. Rats were randomly assigned to six groups: sham-operated group, ischemia/reperfusion group, CS-IVa low-dose group (7.5 mg/kg), CS-IVa medium-dose group (15 mg/kg), CS-IVa high-dose group (30 mg/kg), and diazoxide positive-control group (10 mg/kg). The doses of CS-IVa and diazoxide were selected based on previous cardioprotective studies [9,48]. CS-IVa and diazoxide were each first dissolved in DMSO and then diluted with sterile normal saline to the required working concentrations; the final DMSO concentration did not exceed 1% (*v*/*v*) [49]. Both drugs were administered intraperitoneally at the onset of reperfusion at an injection volume of 10 mL/kg. Rats in the sham-operated and ischemia/reperfusion groups received an equal volume of vehicle solution.

### 4.3. Evaluation of the Cardioprotective Effects of CS-IVa

At 24 h after reperfusion, the cardioprotective effects of CS-IVa were evaluated using echocardiography, hemodynamic analysis, myocardial infarct size measurement, histopathological examination, and biochemical assays. Left ventricular ejection fraction (LVEF) and left ventricular fractional shortening (LVFS) were determined by M-mode echocardiography. Hemodynamic function was assessed using the maximum rates of left ventricular pressure rise and decline (+dP/dt_max_ and −dP/dt_max_). Myocardial infarct size was quantified by Evans Blue/TTC double staining and expressed as the ratio of infarct area to area at risk. Histopathological injury was evaluated by hematoxylin and eosin (H&E) staining. Serum levels of CK-MB, cTnI, LDH, and MDA, as well as serum SOD activity, were determined using commercial assay kits or an automated biochemical analyzer according to the manufacturers’ instructions.

### 4.4. UHPLC-MS/MS Quantification of CS-IVa and Method Validation

Plasma concentrations of CS-IVa were determined using a validated UHPLC-MS/MS method with digoxin as the internal standard. Chromatographic separation was performed on a Waters ACQUITY UPLC BEH C18 column (2.1 × 100 mm, 1.7 μm), and mass spectrometric detection was conducted in negative ion multiple reaction monitoring (MRM) mode. The MRM transitions were *m*/*z* 793.5 → 631.4 for CS-IVa and m/z 779.6 → 649.6 for digoxin. Plasma samples were processed by protein precipitation, centrifugation, evaporation, reconstitution, and injection.

The method was validated according to commonly accepted bioanalytical method validation principles. Good linearity was achieved over 1.22–10,000 nM, with a lower limit of quantification of 1.22 nM. Validation parameters included specificity, precision, accuracy, extraction recovery, matrix effect, stability, and dilution integrity, all of which met acceptable analytical criteria. Detailed chromatographic conditions, sample preparation procedures, MRM parameters, and validation results are provided in Supplementary Methods S2–S4 and Tables S1–S5.

### 4.5. Pharmacokinetic Study and Exploratory Exposure–Response Assessment

In normal rats, CS-IVa was administered intraperitoneally at doses of 7.5, 15, and 30 mg/kg (*n* = 8 per group) to evaluate dose-dependent systemic exposure. Under pathological conditions, MIRI rats received CS-IVa intraperitoneally at 15 mg/kg (*n* = 8). Blood samples were collected over 0–1440 min after administration according to predefined group-specific sampling schedules. Plasma was separated and stored at −80 °C until analysis. Detailed sampling schedules are provided in Supplementary Methods S5.

Non-compartmental pharmacokinetic analysis was performed using Phoenix WinNonlin software (version 8.3; Certara USA, Inc., Princeton, NJ, USA). To evaluate the influence of MIRI on CS-IVa pharmacokinetics, NCA-derived parameters were statistically compared between the Nor-15 mg/kg and MI/RI-15 mg/kg groups using Welch’s independent-samples *t*-test.

CK-MB was selected as the longitudinal pharmacodynamic endpoint for exploratory exposure–response analysis because serial CK-MB measurements were available at time points corresponding to the PK sampling schedule. cTnI and LDH were used as supportive myocardial injury validation biomarkers. The treatment-related pharmacodynamic response was expressed as ΔCK-MB, calculated as the CK-MB level in the untreated MI/RI group minus the CK-MB level in the CS-IVa-treated MI/RI group at each matched time point:

ΔCK-MB(t) = CK-MB_MI/RI_(t) − CK-MB_CS-IVa_(t)

The CK-MB-based exposure–response assessment was interpreted as exploratory because CK-MB is an injury-release biomarker rather than a direct pharmacological target of CS-IVa.

### 4.6. PK Model Re-Evaluation and Robustness Analysis

Candidate PK models for CS-IVa in MIRI rats were re-evaluated using Phoenix WinNonlin software (version 8.3; Certara USA, Inc., Princeton, NJ, USA). To improve model reliability and reduce the risk of overparameterization, one-compartment and two-compartment models with first-order absorption were evaluated, with or without lag time. For each structural model, three weighting schemes were compared: unweighted, 1/Ŷ, and 1/Ŷ^2^, where Ŷ represents the model-predicted concentration. Model selection was based on a comprehensive assessment of goodness-of-fit plots, residual distribution, AIC, SBC, parameter CV%, condition number, convergence behavior, and model parsimony. The full candidate model comparison is provided in Supplementary Table S6.

To further evaluate the robustness of the revised descriptive PK model, a leave-one-rat-out refitting analysis was performed in the MI/RI-15 mg/kg group. The model was first fitted using the full dataset from eight rats and then refitted eight additional times, each time excluding one rat. Model convergence, condition number, and predicted exposure-related parameters were recorded for each refit. Detailed results are provided in Supplementary Table S7.

### 4.7. Exploratory Exposure–Response Analysis

To explore the temporal relationship between plasma CS-IVa exposure and ΔCK-MB response, concentration–effect plots were constructed using plasma concentration and ΔCK-MB values at matched time points. Because a counterclockwise hysteresis pattern was observed, an effect-compartment approach was applied as an empirical exploratory method to describe the temporal delay between plasma CS-IVa exposure and ΔCK-MB response. Ke0 sensitivity analysis was conducted using a series of candidate Ke0 values to evaluate the robustness of the downstream effect-compartment interpretation. The effect-compartment analysis was interpreted as exploratory and was not used as a definitive mechanistic PK/PD model. Detailed procedures and Ke0 sensitivity results are provided in Supplementary Methods S7 and Table S8.

As supplementary exploratory predictive analyses, three data-driven models—a graph attention network (GAT), long short-term memory network (LSTM), and multilayer perceptron (MLP)—were evaluated for ΔCK-MB response prediction. The input feature was normalized plasma CS-IVa concentration, and the output variable was the ΔCK-MB response. The graph representation in the GAT model was defined as a computational association graph for PK–PD observations rather than a biological molecular interaction network. Accordingly, the deep learning results were used only for supplementary prediction and were not used for mechanistic inference or primary PK/PD conclusions. Model performance was evaluated using five-fold cross-validation, with MSE, MAE, and R^2^ as evaluation metrics. Detailed model architectures, training parameters and predictive results are provided in Supplementary Methods S8 and Supplementary Results Figure S4.

### 4.8. Statistical Analysis

Data are presented as mean ± standard deviation (SD). Statistical analyses were performed using SPSS 20.0, and graphical representations were generated using GraphPad Prism 8.0.1. For pharmacodynamic, biochemical, and cardiac functional comparisons among multiple groups, one-way analysis of variance (ANOVA) followed by a Bonferroni post hoc test was used. To evaluate the effect of MIRI on CS-IVa pharmacokinetics, NCA-derived PK parameters between the Nor-15 mg/kg and MI/RI-15 mg/kg groups were compared using Welch’s independent-samples *t*-test. A *p*-value of less than 0.05 was considered statistically significant.

## 5. Conclusions

In conclusion, CS-IVa exerted cardioprotective effects in MI/RI rats, as evidenced by improved cardiac function, reduced myocardial infarct size, attenuated histopathological injury, decreased myocardial injury biomarkers, and improved oxidative stress-related indices. MIRI markedly altered the overall pharmacokinetic behavior of CS-IVa, including delayed absorption-related parameters, reduced systemic exposure, shortened apparent half-life, and altered apparent distribution, indicating that disease-state-related pharmacokinetic changes should be considered in the preclinical evaluation of cardioprotective candidates. The revised descriptive PK model showed improved numerical stability and robustness, while the CK-MB-based exposure–response analysis revealed a temporal dissociation between plasma CS-IVa exposure and pharmacodynamic response. However, because CK-MB is an injury-release biomarker and the effect-compartment analysis was sensitive to Ke0 selection, this exposure–response relationship should be interpreted as exploratory rather than as a definitive mechanistic PK/PD model. Overall, these findings provide a pharmacokinetic and exploratory exposure–response basis for understanding CS-IVa under MI/RI pathological conditions. Further studies incorporating individual-level longitudinal PD endpoints, tissue distribution data, clinically relevant formulations, and translational PK/PD bridging are needed before dosing recommendations can be made.

## Figures and Tables

**Table 1:** Linear range, regression equation, correlation coefficient, and lower limit of quantification of CS-IVa in rat plasma.

Compound	Linear Range (nM)	Regression Equation	Correlation Coefficient (R^2^)	LLOQ (nM)
CS-IVa	1.22–10,000	Y = 0.000042X + 0.000002	R^2^ = 0.9996	1.22

**Table 2:** Non-compartmental pharmacokinetic parameters of CS-IVa in normal and MI/RI rats.

PK Parameters	Nor-7.5 mg/kg (*n* = 8)	Nor-15 mg/kg (*n* = 8)	Nor-30 mg/kg (*n* = 8)	MI/RI-15 mg/kg (*n* = 8)	*p* Value vs. Nor-15
C_max_ (mg/L)	5.38 ± 0.74	9.34 ± 0.52	23.66 ± 2.58	1.52 ± 0.14 ***	<0.001
T_max_ (min)	13.13 ± 2.59	18.75 ± 6.94	37.50 ± 13.89	54.38 ± 7.76 ***	<0.001
AUC_0–t_ (min·mg/L)	353.01 ± 34.18	1527.05 ± 69.42	4635.15 ± 485.94	706.22 ± 19.42 ***	<0.001
AUC_0–∞_ (min·mg/L)	360.99 ± 36.51	1655.36 ± 102.33	4756.53 ± 512.34	717.20 ± 18.81 ***	<0.001
T_1/2_ (min)	326.69 ± 110.07	445.89 ± 32.30	277.11 ± 5.28	238.66 ± 14.91 ***	<0.001
MRT_0–t_ (min)	179.84 ± 18.43	319.76 ± 31.84	352.53 ± 28.54	334.69 ± 9.90	0.24
MRT_0–∞_ (min)	219.91 ± 55.85	455.09 ± 65.71	390.03 ± 32.02	356.94 ± 15.29 **	0.004
V_d_ (L/kg)	9.75 ± 3.10	5.83 ± 0.23	2.55 ± 0.25	7.21 ± 0.54 ***	<0.001
CL (L/min/kg)	0.02 ± 0.00	0.01 ± 0.00	0.01 ± 0.00	0.02 ± 0.00	N.C.

Note: Data are presented as mean ± SD. *p*-values indicate comparisons between the Nor-15 mg/kg and MI/RI-15 mg/kg groups using Welch’s independent-samples *t*-test. ** *p* < 0.01, *** *p* < 0.001 vs. Nor-15 mg/kg. N.C., not calculated.

## Data Availability

The original contributions presented in this study are included in the article/Supplementary Material. Further inquiries can be directed to the corresponding authors.

## Supplementary Materials

The following supporting information can be downloaded at: https://www.mdpi.com/article/10.3390/ph19050749/s1. Figure S1: Candidate PK model fitting and diagnostic plots for CS-IVa in MI/RI rats. The figure shows representative model fitting curves, observed versus predicted concentration profiles, and diagnostic plots used to evaluate candidate pharmacokinetic models. Figure S2: Ke0 sensitivity analysis for exploratory effect-compartment concentration–ΔCK-MB plots. The figure illustrates the relationships between simulated effect-compartment concentrations under different Ke0 assumptions and CK-MB-derived pharmacodynamic responses. Figure S3: Diagnostic plots for the exploratory integrated PK/PD fitting attempt not retained for primary interpretation. The plots show the fitting performance and diagnostic results of the exploratory linked PK/PD model, supporting the decision not to use this analysis as the primary interpretation. Figure S4: Supplementary data-driven predictive analysis of ΔCK-MB response. The figure summarizes the exploratory predictive analysis of CK-MB-derived pharmacodynamic response and related model performance/interpretability results. Table S1: MRM parameters for CS-IVa and digoxin internal standard. The table lists the optimized precursor/product ion transitions and mass spectrometric acquisition parameters used for quantitative LC–MS/MS analysis. Table S2: Intra-day and inter-day precision and accuracy of CS-IVa in rat plasma. The table summarizes measured concentrations, precision, and accuracy at different quality-control levels. Table S3: Matrix effect and extraction recovery of CS-IVa in rat plasma. The table presents the matrix effect and extraction recovery results for CS-IVa and the internal standard under the validated sample-preparation conditions. Table S4: Stability of CS-IVa in rat plasma under different storage and handling conditions. The table summarizes the stability results after short-term storage, long-term storage, freeze–thaw cycles, and autosampler/post-preparative conditions. Table S5: Dilution integrity of CS-IVa in rat plasma. The table presents the accuracy and precision results after dilution of high-concentration plasma samples. Table S6: Candidate PK model comparison. The table compares candidate pharmacokinetic models using fitting performance, model-selection criteria, and diagnostic indicators. Table S7: Leave-one-rat-out robustness analysis. The table summarizes the robustness of key pharmacokinetic parameter estimates after sequential exclusion of individual animals. Table S8: Ke0 sensitivity analysis. The table summarizes the tested Ke0 values and their influence on exploratory effect-compartment concentration–response analyses.
