# Supplementary material for: Pharmacokinetics and Exploratory Exposure–Response Analysis of Chikusetsusaponin IVa in Myocardial Ischemia/Reperfusion-Injured Rats
Source: Pharmaceuticals (Basel). 2026 May 11;19(5):749. doi: 10.3390/ph19050749 (PMC13209573; doi:10.3390/ph19050749)
Supplement: Supplementary file 1 [file pharmaceuticals-19-00749-s001.zip › pharmaceuticals-4254545-supplementary.pdf]

Supplementary materials

Supplementary Methods

S1. Detailed Reagent and Instrument Information

Chikusetsusaponin IVa (CS-IVa) reference standard (purity > 98%, lot number 230115) was purchased from Chengdu Push Biotechnology Co., Ltd. Digoxin reference standard (purity > 99.7%, lot number HY-B1049), used as the internal standard, was purchased from MedChemExpress. Diazoxide (lot number B2303301) was purchased from Aladdin Biochemical Technology Co., Ltd. Methanol and acetonitrile of chromatographic grade (purity ⩾ 99.9%) were purchased from Sigma-Aldrich. The CK-MB assay kit (lot number M13017640) was purchased from Wuhan Huamei Bioengineering Co., Ltd. The rat cardiac troponin I (cTnI) assay kit (lot number 20240130), malondialdehyde (MDA) assay kit (lot number 20231204), and superoxide dismutase (SOD) assay kit (lot number 20231204) were purchased from Nanjing Jiancheng Bioengineering Institute. The lactate dehydrogenase (LDH) reagent (lot number 20231204) was purchased from Shandong Becker Biological Industry Co., Ltd. Ultrapure water was prepared using an Elix Essential 15 (UV) water purification system (Millipore, Milford, MA, USA). Plasma CS-IVa concentrations were quantified using an Agilent 1290 ultra-high-performance liquid chromatography system coupled with an Agilent 6490 triple quadrupole tandem mass spectrometer equipped with an electrospray ionization source (Agilent Technologies, Santa Clara, CA, USA).

S2. UHPLC-MS/MS Analytical Conditions

Chromatographic separation was performed on a Waters ACQUITY UPLC BEH C18 column (2.1 × 100 mm, 1.7 μm). The column temperature was set to 45 °C, the injection volume was 5 μL, and the autosampler temperature was maintained at 4 °C. The mobile phase consisted of double-distilled water (A) and acetonitrile (B) at a flow rate of 0.3 mL/min. The gradient elution program was as follows: 0–2.0 min, 30–65% B; 2.0–2.5 min, 65–95% B; 2.5–3.0 min, 95% B; 3.0–3.1 min, 95–30% B; and 3.1–3.5 min, 30% B.

Mass spectrometric detection was performed using an Agilent 6490 triple quadrupole tandem mass spectrometer equipped with an electrospray ionization source operated in negative ion multiple reaction monitoring (MRM) mode. The drying gas temperature was set to 250 °C, the gas flow rate to 14 L/min, the nebulizer pressure to 20 psi, and the capillary voltage to 4000 V. The optimized MRM transitions and associated parameters for CS-IVa and digoxin are listed in Table S1.

Table S1. MRM parameters for CS-IVa and digoxin internal standard.

Analyte	Precursor ion	Product ion	Dwell	Fragmentor	Collision Energy	Polarity
	(m/z)Q1	(m/z)Q3	Time(ms)	(V)	(V)	
CS-IVa	793.5	631.4	247	380	50	Negative
Digoxin (IS)	779.6	649.6	247	380	40	Negative

S3. Preparation of Standard Solutions and Plasma Samples

CS-IVa and digoxin were accurately weighed and dissolved in DMSO to prepare stock solutions. For calibration curve preparation, the CS-IVa stock solution was serially diluted with 50% methanol to prepare working solutions at concentrations of 1.22, 2.44, 4.88, 9.77, 19.53, 39.06, 78.13, 156.25, 312.5, 625, 1250, 2500, 5000, and 10,000 nM. Matrix-matched calibration standards were prepared by adding 10 µL of the corresponding CS-IVa working solution to 10 µL of blank rat plasma. The digoxin stock solution was diluted with methanol to 10 nM as the internal standard working solution.

For calibration standards, 10 µL of CS-IVa working solution, 10 µL of blank rat plasma, and 180 µL of internal standard working solution were sequentially added to a 1.5 mL microcentrifuge tube. For study plasma samples, 10 µL of plasma sample, 10 µL of 50% methanol, and 180 µL of internal standard working solution were added to a 1.5 mL microcentrifuge tube. After vortex mixing for 3 min, the samples were centrifuged at 12,000 rpm at 4 °C for 30 min. A 160 µL aliquot of the supernatant was transferred to a new tube and evaporated to dryness under a gentle stream of nitrogen. The residue was reconstituted in 80 µL of 50% methanol, vortex-mixed for 3 min, and centrifuged again at 12,000 rpm at 4 °C for 30 min. Five microliters of the resulting supernatant was injected into the UHPLC-MS/MS system for analysis. Stock and working solutions were stored at -20 °C before use.

S4. Bioanalytical Method Validation

S4.1. Specificity

Specificity was assessed using blank plasma, blank plasma spiked with CS-IVa and the internal standard, post-dose plasma samples, and reference solutions containing CS-IVa and the internal standard. Method selectivity was evaluated by examining whether endogenous interference peaks were present at the retention times of CS-IVa and the internal standard.

S4.2. Linearity and Lower Limit of Quantification

Calibration curves were established using matrix-matched standards over the concentration range of 1.22–10,000 nM. The calibration curve was constructed by plotting the peak area ratio of CS-IVa to the internal standard against the nominal concentration of CS-IVa and fitted using weighted least-squares regression with a weighting factor of 1/X. The lower limit of quantification (LLOQ) was defined as the lowest concentration that could be reliably quantified with acceptable precision and accuracy.

S4.3. Precision and Accuracy

Intra-day and inter-day precision and accuracy were evaluated using quality control (QC) samples at four concentration levels: LLOQ (1.22 nM), low QC (20 nM), medium QC (625 nM), and high QC (8000 nM), with six replicates at each level. Precision was expressed as relative standard deviation (RSD), and accuracy was expressed as relative error (RE). Precision and accuracy were considered acceptable when they met commonly accepted bioanalytical validation criteria.

S4.4. Extraction Recovery and Matrix Effect

Extraction recovery and matrix effect were evaluated at three QC levels: low QC (20 nM), medium QC (625 nM), and high QC (8000 nM), with six replicates at each level. Extraction recovery was calculated by comparing the analyte-to-internal standard peak area ratios of extracted QC samples with those of post-extraction spiked samples at equivalent concentrations. The matrix effect was assessed by comparing the analyte response in post-extraction spiked plasma samples with that in neat standard solutions at equivalent concentrations.

S4.5. Stability

The stability of CS-IVa in rat plasma was assessed at concentrations of 20, 625, and 8000 nM under the following conditions: bench-top stability at room temperature for 2 h, autosampler stability at 4 °C for 24 h, three freeze–thaw cycles, and long-term storage stability at -20 °C for 15 days. Stability was considered acceptable when the measured concentrations met predefined bioanalytical acceptance criteria.

S4.6. Dilution Integrity

Dilution integrity was evaluated using QC samples at 20,000, 40,000, and 80,000 nM after ten-fold dilution prior to analysis. The reliability of quantification for samples exceeding the upper limit of the calibration range was verified by assessing the precision and accuracy of the diluted samples.

S5. Pharmacokinetic Sampling Schedule

For the normal pharmacokinetic study, rats received intraperitoneal administration of CS-IVa at 7.5, 15, or 30 mg/kg. For the pathological pharmacokinetic study, MI/RI rats received intraperitoneal administration of CS-IVa at 15 mg/kg. Blood samples were collected over 0–1440 min after administration according to the predefined sampling schedule. Plasma was separated by centrifugation and stored at -80 °C until UHPLC-MS/MS analysis.

Serial CK-MB measurements were obtained at time points corresponding to the PK sampling schedule and were used for exploratory exposure–response analysis. The treatment-related pharmacodynamic response was expressed as ΔCK-MB, calculated as the CK-MB level in the untreated MI/RI group minus the CK-MB level in the CS-IVa-treated MI/RI group at each matched time point.

S6. Candidate PK Model Re-evaluation and Robustness Analysis

Candidate PK models for CS-IVa in MI/RI rats were re-evaluated using Phoenix WinNonlin software (version 8.3; Certara USA, Inc., Princeton, NJ, USA). To improve model reliability and reduce the risk of overparameterization, one-compartment and two-compartment models with first-order absorption were evaluated, with or without lag time. For each structural model, three weighting schemes were compared: unweighted, $1/\hat{Y}$, and $1/\hat{Y}^2$, where $\hat{Y}$ represents the model-predicted concentration.

Model selection was based on a comprehensive assessment of goodness-of-fit diagnostics, residual distribution, AIC, SBC, parameter CV%, condition number, convergence behavior, and model parsimony. The complete candidate model comparison is provided in Table S6.

To further evaluate robustness, leave-one-rat-out refitting was performed in the MI/RI-15 mg/kg group. The revised descriptive PK model was first fitted using the full dataset from eight rats and then refitted eight additional times, each time excluding one individual rat. Model convergence, condition number, predicted C_{max}, predicted T_{max}, and predicted AUC were recorded for each refit. Detailed robustness results are provided in Table S7.

S7. Exploratory Exposure–Response and Ke0 Sensitivity Analysis

To explore the temporal relationship between plasma CS-IVa exposure and CK-MB-derived pharmacodynamic response, plasma concentration–ΔCK-MB plots were constructed using matched time-point data. A counterclockwise hysteresis pattern was used as evidence of temporal dissociation between plasma exposure and the observed CK-MB-derived response.

An effect-compartment approach was applied as an empirical exploratory method to describe this temporal delay. Candidate Ke0 values included 0.1, 0.01, 0.001, 0.0005, 0.0002, 0.0001, 0.00005, and 0.00001 min^{-1}. For each candidate Ke0 value, effect-compartment concentration was generated and used for exploratory concentration–response evaluation. PD fitting status, SSR, AIC, correlation coefficient, and normalized hysteresis loop area were recorded.

The normalized hysteresis loop area was calculated after min–max normalization of effect-compartment concentration and ΔCK-MB using the shoelace formula. For Ke0 values with failed PD fitting, the normalized loop area was reported only as a visual descriptor of hysteresis and was not used to support parameterized PD model selection.

Among the successfully converged candidate Ke0 values, Ke0 = 0.001 min^{-1} showed the best overall fitting performance and was selected as the representative empirical Ke0 value for the main exploratory effect-compartment concentration–ΔCK-MB plot. However, because downstream PD fitting was sensitive to Ke0 selection and failed at several lower Ke0 values, the effect-compartment analysis was interpreted as an exploratory empirical description rather than a definitive mechanistic PK/PD model. Detailed results are provided in Table S8 and Figure S2.

S8. Supplementary Data-Driven Predictive Analysis

As supplementary exploratory predictive analyses, three data-driven models—graph attention network (GAT), long short-term memory network (LSTM), and multilayer perceptron (MLP)—were evaluated for ΔCK-MB response prediction. The input feature was normalized plasma CS-IVa concentration, and the output variable was the ΔCK-MB response. Each paired concentration–response observation was treated as one sample for model training and evaluation.

The graph representation in the GAT model was defined as a computational association graph for PK–PD observations rather than a biological molecular interaction network. Thus, nodes represented PK–PD paired observations, and edges represented computational associations used for prediction. The graph did not represent biological interactions among molecules, targets, pathways, tissues, or organs.

All models were implemented in Python/PyTorch and trained using the Adam optimizer with a learning rate of 0.005. Training was performed for 1200 epochs using mean squared error as the loss function. Model performance was evaluated using five-fold cross-validation, with mean squared error (MSE), mean absolute error (MAE), and coefficient of determination (R^2) as evaluation metrics. The data-driven results were used only as supplementary predictive analyses and were not used for mechanistic inference or to support the primary PK/PD conclusions. Detailed predictive results are shown in Figure S4.

Supplementary Results

S1. Bioanalytical Method Validation Results

The bioanalytical method for quantifying CS-IVa in rat plasma was validated in terms of precision, accuracy, matrix effect, extraction recovery, stability, and dilution integrity. The validation results are summarized in Tables S2–S5.

Table S2. Intra-day and inter-day precision and accuracy of CS-IVa in rat plasma (*n* = 6).

Added CS-IVa (nM）	Intra-day			Inter-day		
	Observed CS-IVa (nM) (Mean±SD)	Precision (RSD,%)	Accuracy (RE,%)	Observed CS-IVa (nM) (Mean±SD)	Precision (RSD,%)	Accuracy (RE,%)
LLOQ (1.22)	1.25±0.02	1.83	2.74	1.25±0.02	2.12	2.78
Low (20)	18.35±1.05	5.74	-8.26	18.51±0.58	3.16	-7.46
Medium (625)	619.62±13.94	2.25	-0.86	622.85±9.46	1.52	-0.34
High (8000)	7992.92±90.99	1.14	-0.09	8005.39±69.85	0.87	0.07

Table S3. Matrix effect and extraction recovery of CS-IVa in rat plasma (*n* = 6).

Added CS-IVa (nM)	Matrix effect (%)	Extraction recovery (%)
Low (20)	111.67	94.14
Medium (625)	95.57	88.19
High (8000)	97.84	95.64

Table S4. Stability of CS-IVa in rat plasma under different storage and handling conditions (*n* = 6).

Added CS-IVa (nM)	25 ℃ for 2 h			4 ℃ for 24 h		
	CS-IVa (nM) (Mean±SD)	Precision (RSD,%)	Accuracy (RE,%)	CS-IVa (nM) (Mean±SD)	Precision (RSD,%)	Accuracy (RE,%)

Low(20)	18.76±0.66	3.49	-6.18	19.09±0.73	3.8	-4.53
Medium(625)	626.70±9.16	1.46	0.27	613.94±7.36	1.2	-1.77
High(8000)	8115.93±117.48	1.45	1.45	8047.68±63.10	0.78	0.6

Added CS-IVa (nM)	Freeze-thaw			Frozen for 15 days		
	CS-IVa (nM) (Mean±SD)	Precision (RSD,%)	Accuracy (RE,%)	CS-IVa (nM) (Mean±SD)	Precision (RSD,%)	Accuracy (RE,%)
Low (20)	19.92±0.68	3.39	-0.41	18.44±0.41	2.21	-7.79
Medium (625)	624.45±6.05	0.97	-0.09	629.20±15.32	2.44	0.67
High (8000)	7971.98±133.22	1.67	-0.35	8032.17±91.89	1.14	0.4

Table S5. Dilution integrity of CS-IVa in rat plasma (n = 6).

Added CS-IVa (nM)	Observed CS-IVa (nM) (Mean±SD)	Precision (RSD,%)	Accuracy (RE,%)
Low (20000)	19097.35±569.41	2.98	-1.93
Medium (40000)	39693.56±1110.97	2.8	-0.77
High (80000)	72375.36±2243.04	3.1	-9.53

S2. Candidate PK Model Comparison and Robustness Results

To support the revised PK model selection, 12 candidate PK models were compared using different structural assumptions and weighting schemes. Model selection was based on goodness-of-fit diagnostics, AIC, SBC, SSR, WSSR, parameter CV%, condition number, convergence behavior, and model parsimony. The complete model comparison is shown in Table S6 and Figure S1.

Table S6. Sensitivity analysis of candidate PK model structures and weighting schemes for CS-IVa in MI/RI rats.

No.	Model structure	Tlag	Weighting	Converged	AIC	SBC	SSR	WSSR	Condition number	Key parameter CV% / stability	Decision
1	1-compartment, first-order absorption	No	Unweighted	Yes	-18.2736	-16.5788	0.154555	0.154555	1.518 x 10^3	V/F 32.18%; K01 40.57%; CL/F 11.33%; Cmax 4.41%; Tmax 7.74%	Selected: best balance of GOF, stability, and parsimony
2	1-compartment, first-order absorption	No	1/Yhat	Yes	-6.5639	-4.8691	0.241470	0.380429	3.198 x 10^2	V/F 13.33%; K01 24.93%; CL/F 11.62%; Cmax 7.32%; Tmax 13.16%	Not selected: poorer information criteria / higher WSSR

3	1-compartment, first-order absorption	No	1/Yhat^2	Yes	5.2320	6.9269	0.417311	0.942636	7.50 x 10^2	V/F 14.04%; K01 27.58%; CL/F 10.31%; Cmax 10.89%; Tmax 18.05%	Not selected: poorer fit
4	1-compartment, first-order absorption	Yes	Unweighted	Yes	-16.2737	-14.0139	0.154554	0.154554	2.732 x 10^3	Tlag CV extremely high (>10^5%); K01 53.36%; CL/F 12.33%; Cmax 4.69%	Not selected: Tlag not identifiable; no meaningful fit improvement
5	1-compartment, first-order absorption	Yes	1/Yhat	Yes	-3.4982	-1.2384	0.218320	0.412931	7.075 x 10^2	Tlag CV 403.40%; K01 37.62%; CL/F 12.50%; Cmax 8.10%	Not selected: unstable Tlag and inferior fit
6	1-compartment, first-order absorption	Yes	1/Yhat^2	Yes	7.6707	9.9305	0.404308	0.974987	1.300 x 10^3	Tlag CV 299.88%; K01 48.71%; CL/F 11.08%; Cmax 11.81%	Not selected: unstable Tlag and poor GOF
7	2-compartment, first-order absorption	No	Unweighted	Yes	-14.4097	-11.5850	0.152945	0.152945	4.168 x 10^6	CL/F 2090.41%; V2/F 8247.91%; CLD2/F 3663.87%; beta 6637.53%	Rejected: severe non-identifiability / overparameterization
8	2-compartment, first-order absorption	No	1/Yhat	Yes	-12.0785	-9.2538	0.169913	0.182985	4.10 x 10^4	V2/F 446.20%; CLD2/F 126.47%; beta 368.94%; B 271.18%	Not selected: improved vs unweighted 2-compartment, but still unstable
9	2-compartment, first-order absorption	No	1/Yhat^2	Yes	-9.0545	-6.2298	0.234327	0.230909	3.202 x 10^6	V1/F 66776.94%; K01 66848.28%; V2/F 8115.96%; CLD2/F 8358.89%	Rejected: severe non-identifiability
10	2-compartment, first-order absorption	Yes	Unweighted	Yes	-12.4563	-9.0179	0.152971	0.152971	4.569 x 10^6	Tlag CV 443082%; CL/F 2397.03%; V2/F 9283.70%; CLD2/F 4129.96%	Rejected: overparameterized and Tlag not identifiable
11	2-compartment, first-order absorption	Yes	1/Yhat	No	--	--	--	--	--	Model did not converge	Rejected: non-convergence
12	2-compartment, first-order absorption	Yes	1/Yhat^2	Yes	-6.9611	-3.5714	0.245233	0.232574	5.447 x 10^3	Tlag CV 9548.90%; A 206.57%; B 95.41%; beta 83.66%; Cmax 7.79%	Not selected: unstable Tlag and poorer fit

Note: GOF, goodness of fit; AIC, Akaike information criterion; SBC, Schwarz Bayesian criterion; SSR, sum of squared residuals; WSSR, weighted sum of squared residuals; CV%, coefficient of variation; Tlag, lag time; $\hat{Y}$, model-predicted concentration. Model selection was based on a joint assessment of GOF diagnostics, residual behavior, AIC/SBC, parameter CV%, condition number, numerical stability, convergence behavior, and model parsimony. The one-compartment model with first-order absorption, no lag time, and unweighted fitting was selected as the revised working descriptive PK model. This model was used for descriptive exposure characterization and exploratory exposure–response analysis rather than as a definitive mechanistic PK model. "—" indicates that the model did not converge or that the corresponding index was not available.

To further assess the robustness of the revised descriptive PK model, leave-one-rat-out refitting was performed in the MI/RI-15 mg/kg group. The model was refitted after excluding one rat at a time, and convergence status, condition number, and predicted exposure-related parameters were recorded. The detailed results are shown in Table S7.

Table S7. Leave-one-rat-out robustness analysis of the revised descriptive PK model.

Dataset	Convergence	AIC	SBC	SSR	WSSR	Condition number	V/F, L/kg (CV%)	K01, 1/min (CV%)	CL/F, L/min/kg (CV%)	AUC, min·mg/L (CV%)	Cmax, mg/L (CV%)	Tmax, min (CV%)	Conclusion preserved
Full data	Yes	-18.2736	-16.5788	0.154555	0.154555	1518	5.4017 (32.18)	0.01518 (40.57)	0.03589 (11.33)	417.99 (11.32)	1.4597 (4.41)	96.81 (7.74)	Yes
Without R1	Yes	-17.1880	-15.4932	0.168016	0.168016	1449	5.1002 (32.48)	0.01532 (40.80)	0.03479 (11.11)	431.11 (11.10)	1.5364 (4.37)	95.18 (7.68)	Yes
Without R2	Yes	-19.2804	-17.5856	0.143037	0.143037	1067	5.9297 (23.84)	0.01717 (32.19)	0.03586 (11.13)	418.35 (11.12)	1.4343 (4.31)	93.84 (7.74)	Yes
Without R3	Yes	-17.1649	-15.4700	0.168315	0.168315	1159	6.1685 (26.73)	0.01645 (36.05)	0.03531 (12.81)	424.77 (12.80)	1.3843 (4.85)	98.42 (8.65)	Yes
Without R4	Yes	-14.1133	-12.4184	0.212848	0.212848	2389	5.2081 (53.46)	0.01323 (63.46)	0.03643 (13.99)	411.75 (13.97)	1.4088 (5.38)	102.23 (9.23)	Yes

Without R5	Yes	-17.1017	-15.4069	0.169135	0.169135	1312	5.3837 (30.52)	0.01601 (39.17)	0.03588 (11.46)	418.02 (11.45)	1.4912 (4.51)	93.78 (7.99)	Yes
Without R6	Yes	-19.4124	-17.7175	0.141593	0.141593	1185	5.6574 (25.69)	0.01653 (33.80)	0.03584 (10.80)	418.49 (10.79)	1.4611 (4.21)	94.05 (7.51)	Yes
Without R7	Yes	-23.0258	-21.3310	0.107232	0.107232	2162	5.0520 (35.83)	0.01377 (42.74)	0.03653 (9.42)	410.64 (9.41)	1.4566 (3.69)	98.49 (6.37)	Yes
Without R8	Yes	-17.4899	-15.7950	0.164159	0.164159	2455	4.7495 (49.69)	0.01336 (57.96)	0.03605 (11.22)	416.05 (11.21)	1.5012 (4.43)	97.97 (7.62)	Yes

Note: The revised descriptive PK model was the one-compartment model with first-order absorption, no lag time, and unweighted fitting. One rat was excluded at a time, and the same model was refitted using the remaining seven rats. Values in parentheses indicate CV%. AUC, area under the concentration–time curve; CL/F, apparent clearance; V/F, apparent volume of distribution. All refits converged successfully, indicating that the revised descriptive PK model was not disproportionately driven by any single animal.

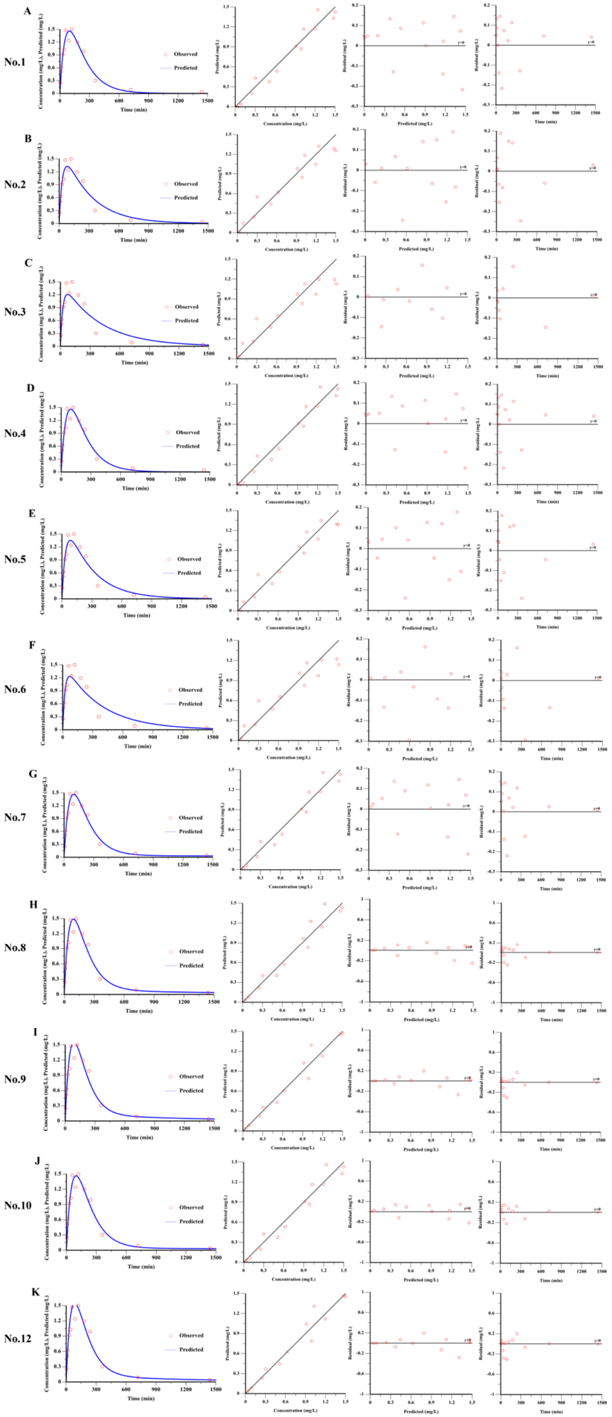
A
No.1
B
No.2
C
No.3
D
No.4
E
No.5
F
No.6
G
No.7
H
No.8
I
No.9
J
No.10
K
No.12
Observed
Predicted
Concentration (mg/L), Predicted (mg/L)
Time (min)
Predicted (mg/L)
Concentration (mg/L)
Residual (mg/L)
y=0

Figure S1. Candidate PK model fitting and diagnostic plots for CS-IVa in MI/RI rats. Representative fitting and diagnostic plots are shown for candidate PK models evaluated during model re-selection. These plots were used together with AIC, SBC, parameter CV%, condition number, convergence behavior, residual distribution, and model parsimony to support the selection of the revised descriptive PK model. The complete candidate model comparison is provided in Table S6.

S3. Ke0 Sensitivity Analysis

Ke0 sensitivity analysis was performed to evaluate the robustness of the empirical effect-compartment interpretation. Candidate Ke0 values included 0.1, 0.01, 0.001, 0.0005, 0.0002, 0.0001, 0.00005, and 0.00001 min^{-1}. The complete results are summarized in Table S8 and illustrated in Figure S2.

Table S8. Ke0 sensitivity analysis for effect-compartment delay characterization.

Ke0 (min^{-1})	t1/2,Ke0 (min)	PD fitting status	SSR	AIC	CORR	Normalized hysteresis loop area	Interpretation
0.1	6.93	Successful	542.950	85.861	-0.515	0.520	Inferior PD fitting
0.01	69.30	Successful	470.721	84.005	0.235	0.385	Poor PD fitting
0.001	693.00	Successful	342.703	79.879	0.565	0.193	Best PD fitting among converged models; empirical delay fit retained cautiously
0.0005	1386.00	Successful	559.616	86.254	-0.676	0.435	Poor PD fitting
0.0002	3465.00	Failed	—	—	—	0.328	PD fitting unstable; area shown only for visual hysteresis comparison
0.0001	6930.00	Failed	—	—	—	0.279	PD fitting unstable; area shown only for visual hysteresis comparison
0.00005	13860.00	Failed	—	—	—	0.252	PD fitting unstable; area shown only for visual hysteresis comparison
0.00001	69300.00	Successful	566.060	86.403	-0.818	0.231	Converged but poor PD fitting; weak correlation

Note: $t_{1/2}$, Ke0 was calculated as 0.693/Ke0. Normalized hysteresis loop area was calculated after min–max normalization of Ce and ΔCK-MB using the shoelace formula. For Ke0 values with PD fitting failure, the normalized loop area is reported only as a visual hysteresis descriptor and was not used to support parameterized PD model selection. Very small Ce values shown in Phoenix screenshots were interpreted according to the scientific notation implied by the corresponding plot scale. Among the successfully converged candidate values, Ke0 = 0.001 min^{-1} showed the best overall fitting performance and was retained only as a representative empirical value for exploratory delay characterization.

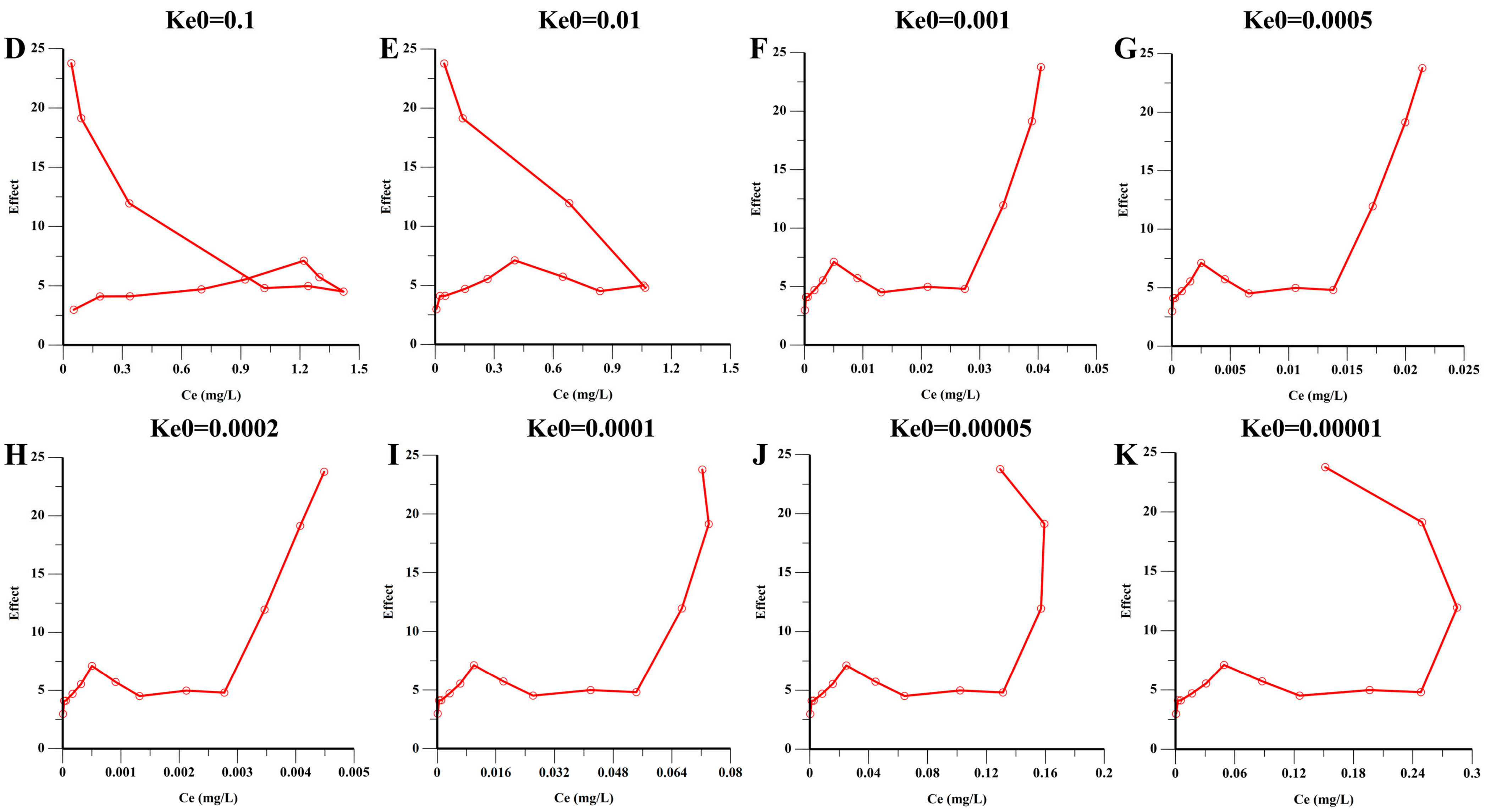

Figure S2. Ke0 sensitivity analysis for exploratory effect-compartment concentration–ΔCK-MB plots. Representative effect-compartment concentration–ΔCK-MB plots were generated using candidate Ke0 values of 0.1, 0.01, 0.001, 0.0005, 0.0002, 0.0001, 0.00005, and 0.00001 min^{-1}. Among the successfully converged candidate values, Ke0 = 0.001 min^{-1} showed the best overall fitting performance and was retained only as a representative empirical value for exploratory delay characterization.

S4. Exploratory Integrated PK/PD Fitting Attempt

An exploratory integrated PK/PD fitting attempt was performed to document whether plasma CS-IVa concentration and the CK-MB-derived response could be simultaneously described within a linked PK/PD framework. The diagnostic plots are shown in Figure S3. Although visual agreement was observed in some panels, this model was not retained for primary interpretation because the revised manuscript focuses on the descriptive PK model and exploratory exposure–response analysis. In addition, CK-MB is an injury-release biomarker rather than a direct pharmacological target of CS-IVa, and Ke0 sensitivity analysis indicated that the downstream PD fitting was sensitive to Ke0 selection. Therefore, this integrated fitting attempt is provided only as supplementary documentation and should not be interpreted as evidence for a definitive mechanistic PK/PD model.

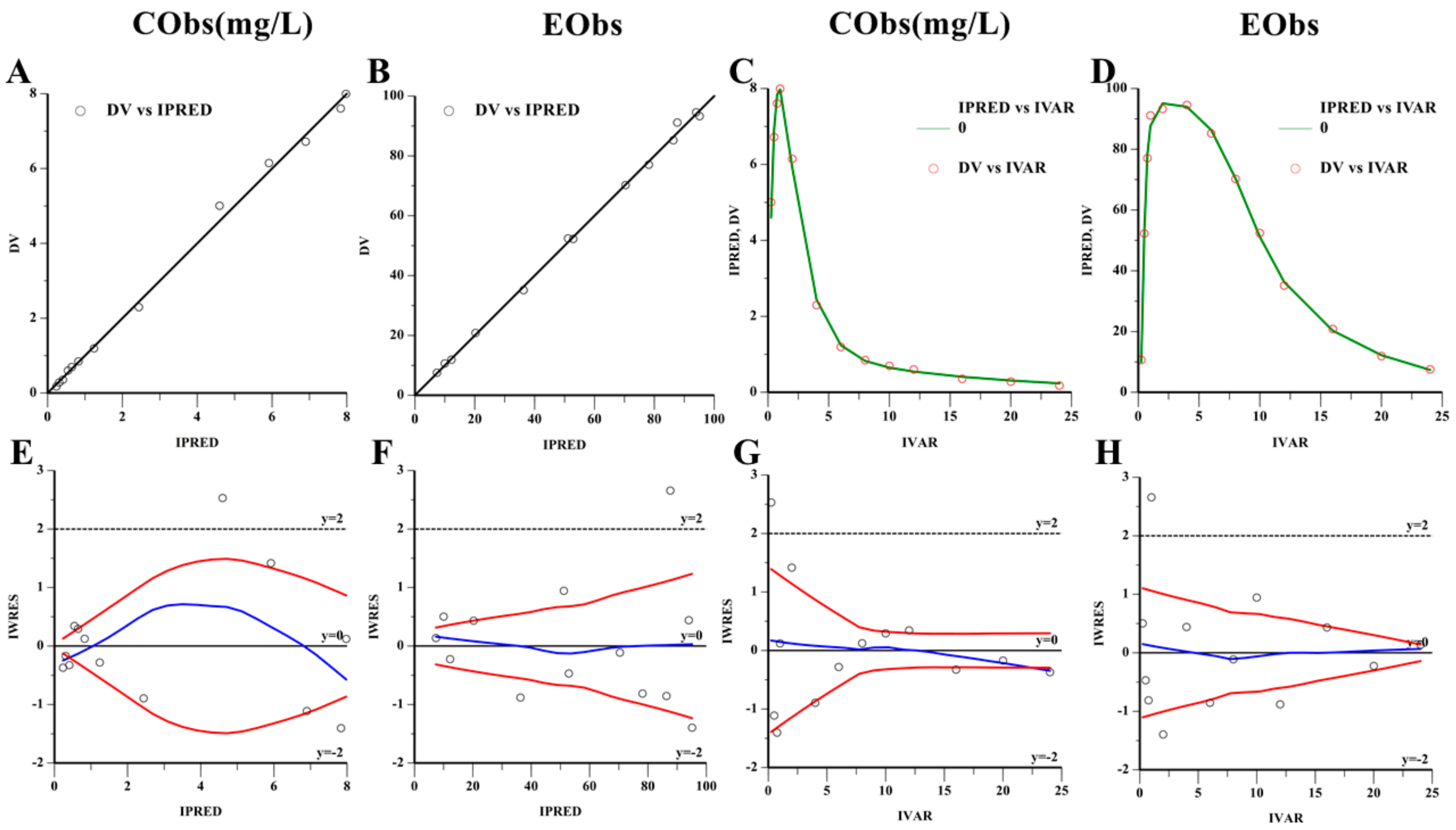

Figure S3. Diagnostic plots for the exploratory integrated PK/PD fitting attempt not retained for primary interpretation. (A) Observed versus individual predicted plasma CS-IVa concentrations, (B) Observed versus individual predicted CK-MB-derived responses, (C) Observed and predicted plasma CS-IVa concentration–time profiles, (D) Observed and predicted CK-MB-derived response–time profiles, (E,F) Individual weighted residuals versus predicted values for plasma CS-IVa concentration and CK-MB-derived response, (G,H) Individual weighted residuals versus time for plasma CS-IVa concentration and CK-MB-derived response. This integrated fitting attempt was included only as supplementary documentation and was not used to support the primary conclusions or dosing recommendations.

S5. Supplementary Data-Driven Predictive Results

As supplementary exploratory predictive analyses, GAT, LSTM, and MLP models were evaluated for ΔCK-MB response prediction. These models were used only as data-driven predictive tools and were not used for mechanistic inference or to support the primary PK/PD conclusions.

Among the models compared, the GAT model showed the best predictive performance within the current dataset, with lower MSE and MAE and higher R^2 than LSTM and MLP. However, given the limited sample size, group-level PD response, and computational nature of the graph representation, these findings should be interpreted cautiously as supplementary predictive results rather than definitive mechanistic conclusions. The graph in the GAT model represents computational associations among PK–PD paired observations, not biological interactions among molecules, targets, tissues, or organs.

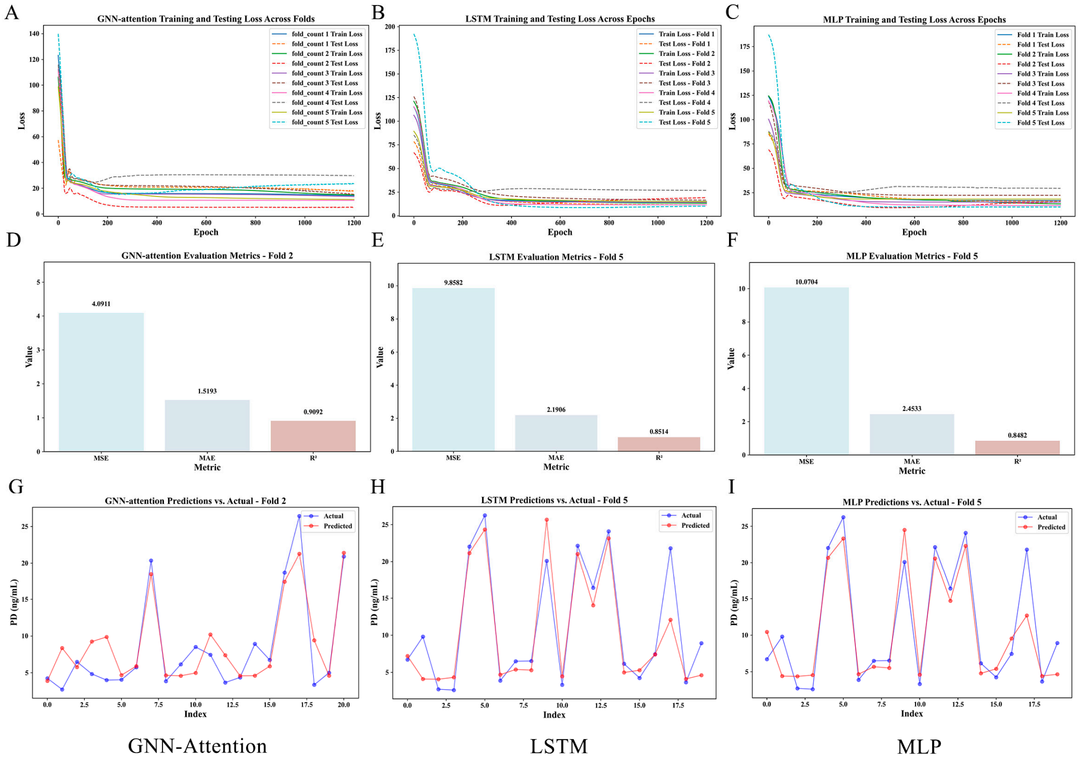

Figure S4. Supplementary data-driven predictive analysis of ΔCK-MB response. (A–C) Training and testing loss curves of the GAT, LSTM, and MLP models across epochs. (D–F) Predictive performance metrics, including MSE, MAE, and R^2, for the GAT, LSTM, and MLP models. (G–I) Observed-versus-predicted ΔCK-MB response plots for the GAT, LSTM, and MLP models. These models were evaluated only as supplementary exploratory predictive tools and were not used for mechanistic inference or to support the primary PK/PD conclusions.
